# The Impact of Education on Weight Loss in Overweight and Obese Adults

**Published:** 2013-09-01

**Authors:** Mohammad Ali Ostovan, Mohammad Javad Zibaeenezhad, Hassan Keshmiri, Shahnaz Shekarforoush

**Affiliations:** 1 Cardiovascular Research Center, Shiraz University of Medical Sciences, Shiraz, IR Iran; 2 Department of Physiology, Islamic Azad University, Arsanjan Branch, Arsanjan, IR Iran

**Keywords:** Weight Loss, Lifestyle, Diet, Physical Activity

## Abstract

**Background:**

Prevalence of obesity is rapidly rising. To reverse the obesity epidemic, efforts should be made to incorporate intensive weight loss programs into medical practice. The primary aim of this study was to change the behavior for achieving a mean weight loss of 5-10% of initial body weight over 6 months in overweight and obese adults.

**Methods:**

In this quasi-experimental study, 266 out of 533 subjects screened for coronary heart disease risk factors in Shiraz Healthy Heart House were overweight or obese. 140 individuals with BMI≥25 completed this study’s 6 month program. The subjects were visited on day 1 and at 2 week intervals and taught intensive lifestyle modification. The subjects who did not lose 5% of their initial body weight after 3 months were assigned to receive 120 mg orlistat three times daily for 3 months in addition to counseling sessions. The main outcome measures were body weight and BMI.

**Results:**

The mean weight and BMI of participants were 78.6±10.7 kg and 30±0.2 kg/m2, respectively. Women included 58% of the sample. 110 subjects (78.5%) lost ≥5% of their initial body weight during 3 months. The Mean weight and BMI loss in these subjects were 7.6±0.8 kg and 2.4±0.3 kg/m2, respectively.

**Conclusions:**

Teaching of how to modify lifestyle and to gain more self-control with eating have the major role in reducing weight and BMI. So, training accompanied by continual follow up for performing the instructions could lead to favourable results.

## 1. Background

Obesity is a well-established risk factor for premature death (1, 2). An increase in visceral fat accounts for many of the metabolic abnormalities (3, 4). The prevalence of obesity and overweight in IR Iran is rising. The excessive consumption of high-calorie foods and sucrose-enriched beverages associated with sedentary lifestyle are the main factors contributing to prevalence of obesity (5). The most successful treatment programs apply a behavior-therapy model which integrates improved nutrition and proper eating habits, regular exercise and lifestyle modification (6). In the first study examining the efficacy of an intensive lifestyle intervention on weight loss in the severely obese adults, the participants with a BMI>40 kg/m2 lost 10% of their initial body weight at 1 year (7).

Despite the steady progress in the management of obesity, its prevalence continues to rise. Considerable research on effective treatment options is required to optimize management and to prevent the increasing prevalence of overweight and obesity. This study aimed to evaluate the weight and BMI loss over 6 months in outpatients to follow intensive lifestyle instructions.

## 2. Patients and methods

According to the Canadian clinical practice guideline (CPG), in this quasi-experimental study (pre-post design), 266 out of 533 subjects screened for obesity in Shiraz Healthy Heart House were overweight (33.08% with BMI≥25 kg/m2) or obese (17.82% with BMI≥30). Exclusion criteria for the study included pregnancy, secondary obesity, drug abuse, thyroid disease and mal-absorption. In addition, all those who failed to appear for follow-up and further consultations and adhere to a diet and more exercise were excluded from the study. A total of 140 individuals with BMI≥25 completed the study’s 6 month program. We sought consent from all participants before conducting the study.

Subjects were visited on day 1 and at 2 week intervals during 6 months. On day 1, the participants were taught weight loss related- behaviour change and instructed how to record them. Lifestyle counselling was delivered by a dietician, exercise specialist and physician in individual sessions. Height was measured once. Further visits began with a weighing and then a review of participants’ recording of food intake, physical activity, and other behaviors in sequential handouts.

The main goal was to achieve a mean loss of 5-10% of the baseline weight. Diet was written somehow to decrease energy intake by 500-1000 kcal per day. Energy intake was calculated 25 kcal per kilogram of body weight. The base of dietary therapy in obese patients was on a low-carbohydrate diet and DASH Diet protocol with a diet enriched in vegetables, fruits, low-fat, low-fat dairy and grain. In this regimen, high consumption of fibre, potassium, calcium, magnesium and sodium reduction is considered.

The participants were also recommended to change their lifestyle activity, for instance walking rather than riding, using stairs rather than elevators, and avoiding sedentary activities.

Increased physical activity (PA) was recommended for 3-5 times a week, 30-45 minutes each time depending on the gender and BMI. Contraindications to moderate PA were assessed by the Physical Activity Readiness Questionnaire (PAR-Q). The participants were assessed for PA levels and patterns by Physical Activity Questionnaire. Then, they received a 9-week PA program. It varied from controlled individualized exercise programs to informal group sessions coupled with behavior therapy and dietary changes. The subjects received standard vitamin supplements throughout 6 months of the study.

To avoid forgetting the subjects taught, the subjects were provided with an educational pamphlet about diet and increased physical activity. The subjects who did not lose 5% of their initial body weight after 3 months were assigned to receive 120 mg orlistat three times daily for 3 months in addition to counseling sessions (orlistat group). During the study to detect possible side effects of the drug, the liver enzymes were measured.

The variables measured included: 1- height and weight to calculate BMI at baseline, 3 and 6 months; 2- sex and age.

### 2.1. Statistical Analyses

All data were analysed using SPSS version 17.0. Quantitative variables are expressed as mean±SD. Statistical analyses were performed using repeated measure ANOVA, Tuckey post hoc test and dependent t-test. The chi-square test was used for comparisons of qualitative data. A P value less than 0.05 was considered as statistically significant.

## 3. Results

The participants included 81 females (58%) and 59 males (42%), with a mean body weight of 78.6±10.7 kg and a mean BMI of 30±0.2. Table 1 shows mean weight and BMI at the baseline, 3 and 6 months in both sexes.

**Table 1.** Mean Weight (Kg) and BMI (Kg/m2) at Baseline, 3 and 6 Months After Intervention in Both Sexes

Sex	N	Weight				Body mass index(BMI)			
		Baseline	3 months	6months	P value	Baseline	3 months	6months	P[Table-fn fn5662]value
**Male**	59	81.2 ± 1.6	76.9 ± 1.2*	73.0 ± 11.5*	< 0.001	29.9 ± 2.9	28.6 ± 2.8*	27.3 ± 2.6*	< 0.001
**Women**	81	76.9 ± 9.2	73.5 ± 8.8*	69.2 ± 8.0*	< 0.001	30.5 ± 3.0	29.3 ± 3.1*	27.9 ± 2.8*	< 0.001
**Total**	140	78.6 ± 10.7	74.9 ± 10.4*	70.8 ± 9.8*	< 0.001	30.3 ± 2.5	29.0 ± 3.0*	27.6 ± 2.8*	< 0.001

mean±SD, repeated measure ANOVA was used; *significant level vs. baseline

110 subjects (78.5%) lost ≥5% of their initial body weight during 3 months. Of the remaining 30 subjects who received orlistat, 7 dropped out due to medication side effects including flatus, diarrhea, abdominal pain and fecal urgency. Table 2 shows that orlistat combined with counselling caused more weight and BMI loss compared with counselling alone. At the end of study, significantly more subjects in the orlistat group (94.7%) lost 5 percent or more of their initial weight than did those in the counselling group (79.4%) (P<0.001).

**Table 2.** Mean Weight (Kg) and BMI (Kg/m2) at Baseline, 3 and 6 Months After Intervention in Both Groups

Group	N	Weight				Body mass index (BMI)			
		Baseline	3 months	6months	P[Table-fn fn5663]value	Baseline	3 months	6months	P value
**Counselling**	117	77.0±10.2	73.1±9.7*	69.4±9.4*	<0.001	29.6±2.6	28.3±2.5*	27.1±2.3*	<0.001
**Orlistat**	23 87	2±9.5	84.3±9.0*	77.9±8.8*	<0.001	33.6±2.6	32.5±3.0*	30.2±3.3*	<0.001
**Total**	140	78.6±10.7	74.9±10.4*	70.8±9.8*	<0.001	30.3±2.5	29.0±3.0*	27.6±2.8*	<0.001

mean±SD, repeated measure ANOVA was used; *significant level vs. baseline

**Figure 1.**
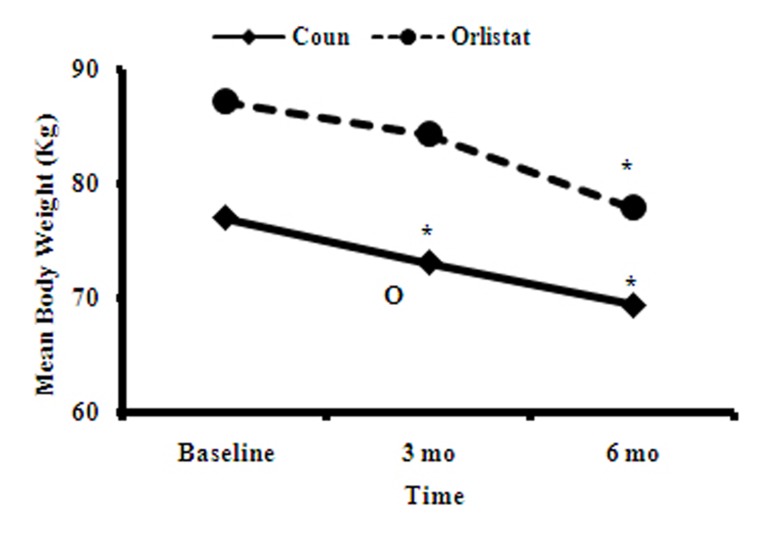
Mean Body Weight at Baseline, 3 and 6 Months in the Counselling and Orlistat Groups *P<0.001 vs. baseline

Mean weight loss at the end of 3 and 6 months was 3.7±0.15 and 7.6±0.8 kg compared with baseline weight, respectively (P<0.001 and 0.001). Mean weight loss in the orlistat group was 9.3±1.8 kg (P<0.001 compared with baseline weight) (Figure 1). Mean weight loss in women during 6 months of follow up was insignificantly more than that in men (10% compared to 8%, respectively).

**Figure 2.**
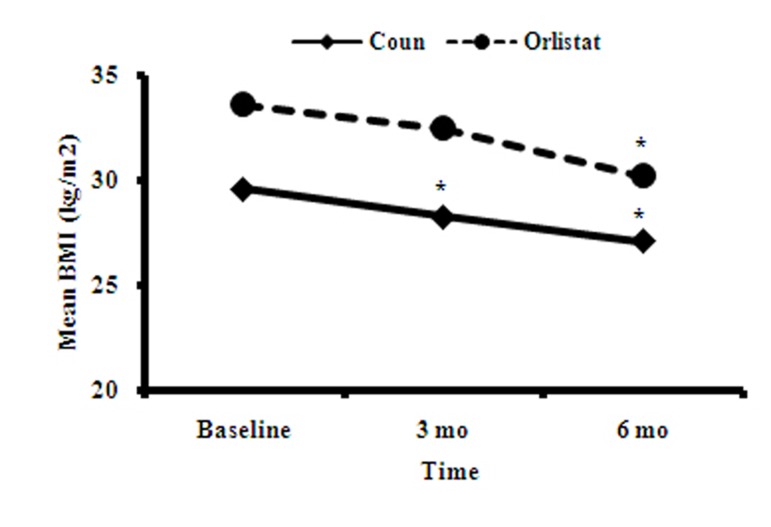
Mean BMI at Baseline, 3 and 6 Months in the Counselling and Orlistat Groups *P<0.001 vs. baseline

Mean BMI loss at the end of 3 and 6 months was 1 and 3 Kg/m2, respectively compared with the baseline BMI (P<0.001). Mean BMI loss in the orlistat group (3 months after consumption) was 3.4 compared to a mean loss of 2.4 kg/m2 for the counselling group. This difference was significant (P<0.0001) (Figure 2).

Gradient of BMI and weight loss was insignificantly more among the women compared with the men (P=0.34 and 0.31, respectively).

Orlistat had no adverse effect on SGOT and SGPT level at the end of the study.

## 4. Discussion

This is one of the few studies in IR Iran focusing on the training of lifestyle modification among overweight and obese subjects. The principal finding of this study was that a large percentage of participants (78.5%) who received intensive lifestyle counselling with follow up lost 5% or more of their initial weight within 3 months, an amount of weight loss that improves obesity-related risk factors, including lipid concentrations, blood pressure, and glycemic control (8-10).

Six months of therapy is accepted as a reasonable period for a 10 percent reduction in body weight. After 6 months, the rate of weight loss usually decreases and the weight plateaus because of a lesser energy wasting at the lower weight (11).

In this study, 33.08% of the subjects were overweight (BMI, 25 to 29.9 kg/m2) and 17.82% were obese (BMI≥30 kg/m2); the prevalence was higher in women. The prevalence of obesity in IR Iran has reached epidemic proportions and is specifically affecting women. In a study on Lipid and Glucose Study in Tehran, 40% of the study population in Tehran, IR Iran, were overweight and 23.1% of them were obese (5). The prevalence of overweight and obesity in Canada is 59% and 23%, respectively (12).

In this study, the intensive lifestyle counselling produced an average weight loss of 7.6 kg at the end of the study, which was greater than that observed in other studies. Wadden et al randomly assigned 390 obese adults to one of three types of intervention. The results showed that the mean (±SE) weight loss with usual care, brief lifestyle counselling, and enhanced brief lifestyle counselling was 1.7±0.7, 2.9±0.7, and 4.6±0.7 kg, respectively. The initial weight decreased at least 5% in 21.5%, 26.0%, and 34.9% of the participants in the three groups, respectively (13).

The results of this study showed that the effects of lifestyle modification and medication would be additive. Wadden et al found that the combination of lifestyle-modification counselling and pharmacotherapy resulted in an average loss of 12.1 kg at one year (14).

The best strategy to prevent obesity is healthy lifestyle, with special emphasis on a balanced healthy diet, adequate physical activity and regular aerobic exercise, maintenance of ideal body weight, and weight reduction in overweight people (5).

In summary, intensive lifestyle counselling helps more than two thirds of overweight or obese participants who attended individual sessions regularly to achieve a significant loss of weight, which may indicate the usefulness of these sessions. Adding orlistat was useful to produce clinically meaningful weight loss for treatment of obesity in few subjects with BMI more than 30.

It is concluded that training accompanied by continual follow up for performing the instructions can lead to favourable results.
